# A Hybrid TDMA/CSMA-Based Wireless Sensor and Data Transmission Network for ORS Intra-Microsatellite Applications

**DOI:** 10.3390/s18051537

**Published:** 2018-05-12

**Authors:** Long Wang, Yong Liu, Zengshan Yin

**Affiliations:** 1 Shanghai Institute of Microsystem and Information Technology, Chinese Academy of Sciences, Shanghai 200050, China; yong.liu@mail.sim.ac.cn (L.Y.); yinzsh@mail.sim.ac.cn (Z.Y.); 2 University of Chinese Academy of Sciences, Beijing 100049, China; 3 Shanghai Engineering Center for Microsatellites, Chinese Academy of Sciences, Shanghai 201210, China

**Keywords:** ORS, wireless, modular, satellite, COTS, TDMA/CSMA, CCA, adaptive slot allocation, Markov model

## Abstract

To achieve launch-on-demand for Operationally Responsive Space (ORS) missions, in this article, an intra-satellite wireless network (ISWN) is presented. It provides a wireless and modularized scheme for intra-spacecraft sensing and data buses. By removing the wired data bus, the commercial off-the-shelf (COTS) based wireless modular architecture will reduce both the volume and weight of the satellite platform, thus achieving rapid design and cost savings in development and launching. Based on the on-orbit data demand analysis, a hybrid time division multiple access/carrier sense multiple access (TDMA/CSMA) protocol is proposed. It includes an improved clear channel assessment (CCA) mechanism and a traffic adaptive slot allocation method. To analyze the access process, a Markov model is constructed. Then a detailed calculation is given in which the unsaturated cases are considered. Through simulations, the proposed protocol is proved to commendably satisfy the demands and performs better than existing schemes. It helps to build a full-wireless satellite instead of the current wired ones, and will contribute to provide dynamic space capabilities for ORS missions.

## 1. Introduction

To accomplish flexible and rapid response tactical missions, the Operationally Responsive Space (ORS) was proposed by the United States Department of Defense (DoD) [1]. Through the implementation of the launch-on-demand of dedicated platforms, the ORS system provides rapid, flexible and economic access to future space missions, including tracking, data relay and Earth observation [2]. To deploy various types of tasks, a common interface is required for integrating different satellite payloads and subsystems [3]. As the main technical verfication project of the ORS, the TacSat plan aims to build a standardized bus and achieve rapid spacecraft design. Since 2006, dozens of satellites have been launched successfully [4,5,6,7,8,9]. Likewise, in China, two mapping satellites, Kuaizhou-1 (KZ-1) and Kuaizhou-2 (KZ-2), have been developed. The missions aim to provide information support for rapid-response land observation, including emergency monitoring and disaster relief [10,11].

The rapid ground integration, assembly and test (AIT) of satellites are significantly crucial yet very challenging in developing an ORS system. Besides, the miniaturization and reliability of such systems are in high demand. In the past decades, with the rapid development of integrated electronic technology, both harness reduction and reliability improvements were accomplished. However, previous studies have shown that cables, interfacing hardware and connectors account for 7~10% of the mass of a satellite, half of which are data transfer cables [12,13]. It has also been revealed that these interdependent physical links tend to increase the redundancy of a satellite, which, in turn, decreases the reliability of the satellites. These issues have been a major obstacle that has hindered the miniaturization and rapid design of ORS satellites [14,15]. Wireless buses and interfaces have been considered as promising alternatives to solve the abovementioned problem. They allow one to eliminate the cables so that both the data rate and reliability of the intra-satellite network can be improved [12,16].

Among many wireless technologies, Optical Wireless (infrared-IR) and Radio Frequency (RF) are leading candidates. Proposed by National Institute for Aerospace Technique of Spain (INTA), the Optical Wireless Links for intra-Spacecraft communications (OWLS) approach has been studied by many groups in the past decades [17,18]. OWLS offers a direct access to high data rate long range communication without any electromagnetic interference/compatibility (EMI/EMC) concerns [18]. However, for optical wireless interfaces, line-of-sight (LOS) communication requires a clear signal path, which imposes limitations on the layout design of satellite cabins. In another case, the diffuse communication will result in a short range and unguided transmission. In addition, according to the CCSDS and other previous studies [19,20], the optical link for intra-spacecraft communications can be easily shielded too.

In contrast, the Radio Frequency (RF) technology is more mature, and it also has better penetration and simpler components. It provides a high data rate without direct LOS. To put it simply, RF wireless interfaces is are flexible, and can be easily monitored. They can effectively simplify and shorten the AIT process of the satellite design. In recent years, RF has therefore been introduced to intra-satellite communication. At Delft University and Surrey University, Zigbee and Bluetooth were directly applied to satellite on-board wireless protocol design [12,21,22,23,24,25], including a wireless digital Sun sensor [23], an EADS micropack wireless temperature transducer [24] and a wireless satellite Delfi-C3 [25]. In Spain, a Zigbee-based hardware architecture for intra-satellite communication was proposed, and its robustness to the space electromagnetic environment was discussed [8]. In Japan [26], ultra-wideband (UWB) was considered as a potential choice for intra-satellite data connections, and several on-board sensing and data communication networks were proposed [27,28,29].

In this article, we study the internal communication system for ORS micro-satellites. We show that with the application of COTS devices in satellite design, data rates can be ramped up to hundreds of Kbps. In particular, compared with other on-board subsystems, the attitude and orbit control subsystem (AOCS) requires lower latency and higher sampling rates. Thus, time-critical communication is ensured. Besides, the number of nodes in the network will be dozens, and the network size is time-varying during different stages of the task. Athough Zigbee has been applied in several on-board sensor networks [14,30,31], it only provides a finite data rate up to 250 Kpbs which is much lower in real application [32]. As an alternative to the wired bus, it is more suitable for applications in pico-satellites and nano-Satellites, which require low data rate intra-spacecraft communication as defined by CCSDS [18]. On the other hand, a limitation of Bluetooth technology lies in the network scale. Usually, the maximum number of nodes in a Bluetooth network is seven. Techniques to increase the nodes number are available, however, they add additional complexity to the network [33].

To briefly summarize, the existing protocols are not appropriate for our project. Therefore, a dedicated wireless protocol needs to be developed. It is desired that such protocol provide an average data rate around 500 Kbps and meet the data delay requirements of the various on-board subsystems. Thus, we present an Intra-Satellite Wireless Network (ISWN), by which both wireless sensor and data transmission are accomplished. Using COTS components, we show that both the complexity and cost of the satellite are reduced, which ultimately increases its reliability [34]. A hybrid TDMA/CSMA protocol is proposed. With an improved channel assessment method and adaptive slot allocation mechanism, the hybrid protocol is expected to improve performance while saving energy.

The article is organized as follows: Section 2 introduces the concept and structure of the ISWN. In Section 3, the physics layer hardware design is presented. Requirements analysis and scheme details of the MAC protocol are proposed in Section 4. Then, a Markov model and related calculations are given in Section 5. In Section 6, the simulations are carried out and discussions are presented. At the end, conclusions are given in Section 7.

## 2. Concepts and Structure

**Figure 1:**
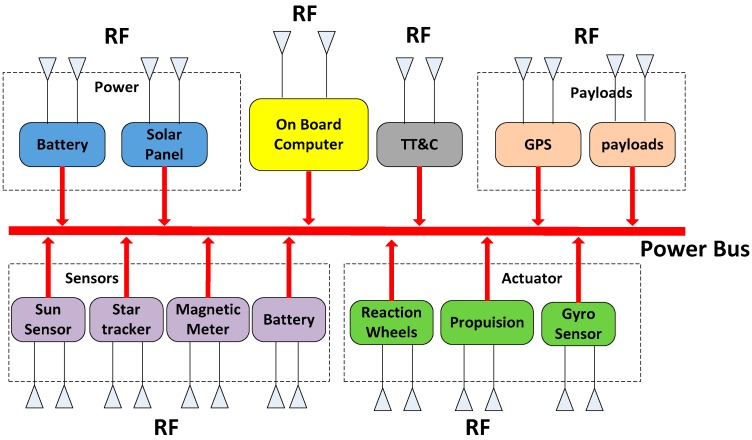
The structure of the ISWN.

To achieve modularization and standardization in ORS satellite design, the satellite is composed of modular cabins that have certain computing ability. Wire connections are eliminated except for the power line. All the data exchanges during the modules are accomplished via a wireless network. The structure of the ISWN is shown in Figure 1 below.

**Figure 2:**
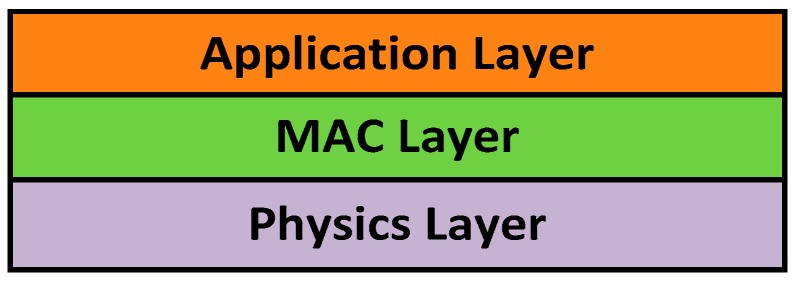
Model of the protocol.

As shown in the figure, the satellite is made up of dozens of RF communication nodes. The base node consists of a central processer CPU and RF communication chips (RFIC), which complete both radio communications and failure diagnosis. Compared to AISC circuits, the application of COTS devices can effectively reduce the time and cost during the AIT phase of the satellite design, which meets the flexibility requirement of the ORS mission. As shown in Figure 2, a simplified model of the seven layers OSI is presented, where the intra-satellite wireless sensor and data transmission network is separated into three layers. In this article, we mainly focus on the physics layer and the MAC layer.

## 3. Physics Layer

**Figure 3:**
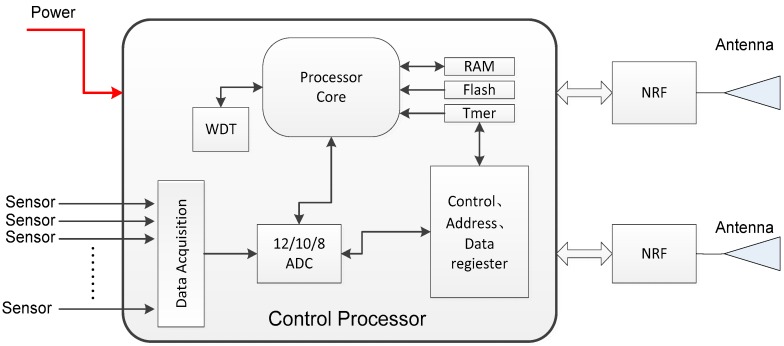
Structure of the wireless nodes.

To facilitate on-board application, careful consideration needs to be taken regarding reliability and low power consumption. In our design, each node consists of one control processor and two RF circuits (RFICs). As a mature commercial circuit, an 8051 series microprocessor from Cygnal (Austin, TX, USA) is adopted as the control processor. Another chip from Texas Instruments (TI) (Dallas, TX, USA) is chosen as the data transceiver, which supports three types of modulation, FSK, MSK and GFSK. The chip supports configurable Clear Channel Assessment-Energy Detect (CCA-ED). This provides the possibility of using contention-based MAC protocol design. In addition, the microprocessor has been verified on-orbit by the Chinese satellite Shiyan-3 and functioned well [35]. The structure of the wireless node is illustrated in Figure 3 below.

In Figure 3, each node consists of one control processer and two RF circuits. A physical address (hardware address) is used as the identification marker of each RF component. By setting the physical address, each node supports full-duplex communication, in which one RFIC is used as transmitter while the other as a receiver. In the case of function failure of any RFIC, the surviving RF chip can work alternatively of transmitter and receiver. Working in the 2.4~2.5 GHz ISM band, the chip’s data rate is 500 Kbps. The main parameters of the wireless node are given in Table 1.

## 4. MAC Protocol Design

### 4.1. Requirements Analysis

The ISWN is expected to complete both on-board sensing and subsystem data transmission, thus the data demands should be considered seriously. According to the data service requirements, the on-board data can be classified into three categories, as shown in Table 2.

Let $\;N_{A}$, $N_{B}$ and $N_{C}$ be the number of nodes in each category. The first two kinds of nodes are expected to have high data rate or strict packet delay requirements. Thus, guaranteed slots with a center controlled scheduling scheme are more suitable. In contrast, the last kind of nodes have loose requirements both in time delay and data rates, which means that they are tolerant to contention access. To summarize, the requirements are as follows:

- Enough data rate for both payloads and subsystems;
- Delay performance guarantee for the time critical subsystems, such as AOC subsystem;
- Simple structure and support rapid development;
- Robustness and fault tolerance;
- Energy efficiency, since the available on-board power resources are limited.

### 4.2. Hybrid TDMA/CSMA

As mentioned earlier, the ISWN is expected to complete both on-board sensing and subsystems’ data transmission, which leads to expanded scale and diversified data requirements. Especially, for the on-board AOC system, the delay performance of these nodes should be strictly maintained. Time-division multiple access (TDMA) and carrier sense multiple access with collision avoidance (CSMA/CA) are regarded as two possible solutions for multi access method. Usually, the CSMA/CA provides a simple, distributed, burden-less scheme for adaptive traffic, while the TDMA method offers a centralized, scheduled scheme, in which the target QoS can be guaranteed by resource assignment.

In a CSMA/CA network, it is difficult to ensure the delay performance especially when the network is large in scale. In addition, the packet delay will increase rapidly with the increasing traffic load, which may lead to difficulties in real-time attitude and orbit control. Thus, CSMA/CA is not a suitable choice for an intra-satellite network, which contains dozens of nodes. As for TDMA, a fixed slot assignment-based TDMA network presents stable packet delay and requires very accurate duty cycling. Compared to the CSMA/CA, it increases the communication cycle when the network traffic is low, since every node must wait until its turn to communicate.

To meet a variety of requirements, the MAC protocol is expected to have the characteristics of combing TDMA with CSMA/CA. Hybrid protocols have been applied in some scenarios, such as vehicular ad-hoc networks ([HTC-mac [36] and Her-mac [37] and wireless sensor networks (Z-MAC [38], Tree-MAC [39] and MCLMAC [40], etc.), and they have been proved to achieve improved throughput and reduced control overhead. They mainly aim to accomplish efficient access for multi-hop communication or/and mobile nodes.

**Figure 4:**
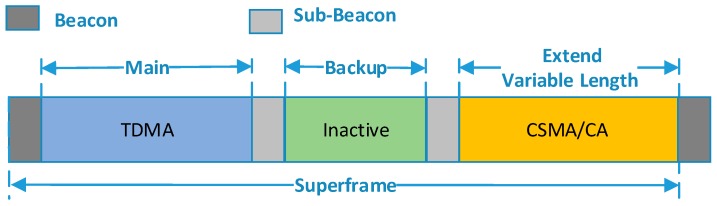
Structure of the packet superframe.

In an on-board wireless network, the location and the number of network nodes are fixed, and only one-hop communication is involved, so its topology and data requirements are different from either of them. Thus, a new hybrid TDMA/CSMA scheme for the intra-satellite network is proposed here. As an on-board network for intra-satellite, a star topology is adopted, in which the data is periodically transmitted. The on-board computer (OBC) is set as the master node, while other subsystems are set as the slave nodes. In traditional wired satellites, the synchronization was achieved with a plus-per-second (PPS) mechanism, in which the time information flows were transmitted by a wired data bus. Similarity, in this work, the synchronization between the OBC and other nodes is achieved by radio beacons. The network works in a periodic mode, where both center controlled scheduling and contention access are adopted. The network communication cycle is divided into three periods:

1. TDMA-based Main Communication period (MCP);
2. Backup communication period (BCP);
3. CSMA/CA-based Extended Communication period (ECP);

The main communication phase provides center scheduled slots which are assigned by the nodes’ ID. Nodes in type A and type B will communicate in this period. By contrast, the extended communication phase provides contention-based slots, in which an improved slotted CSMA/CA strategy is performed and nodes in type C will transmit their packet in this period. Due to the orbital characteristics, the on-board applications are periodic, thus we assume the number of the active nodes changes periodically too. Since the available power of a satellite is limited, a fixed-length superframe with a variable-length CSMA period was adopted. Accordingly, a variable-length inactive period is applied to the back-up communication period. In the inactive period, all nodes turn off their radio to conserve energy. Two sub-beacons are broadcasted by the host node to mark the boundary between adjacent periods. The structure of the superframe is shown in Figure 4.

**Figure 5:**
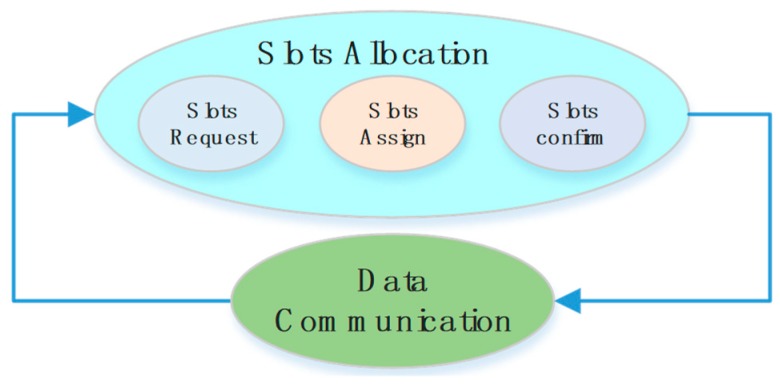
Slot scheduling process in the TDMA period.

According to the data requirements, nodes in type A and type B requires scheduled slots to achieve high data rate and/or low latency. Thus, their data transmission will be performed during the MCP. In this period, nodes obtain their slots by center controlled scheduling. To complete the slot allocation, a three-way handshake process is performed, and after the slots information will be stored both in the host node and the slave node. The slot allocation process for different nodes is staggered by their device id, the slot request frame, assign frame and confirm frame are transmitted at a particular slot sorted by their ID value. In addition, slots will be recovered when a node goes into the sleeping state. Once the node wakes up, the slot allocation process will be executed again. By doing so, dynamic slot allocation and plug-and-play can be achieved. The slot allocation process is shown in Figure 5.

**Figure 6:**
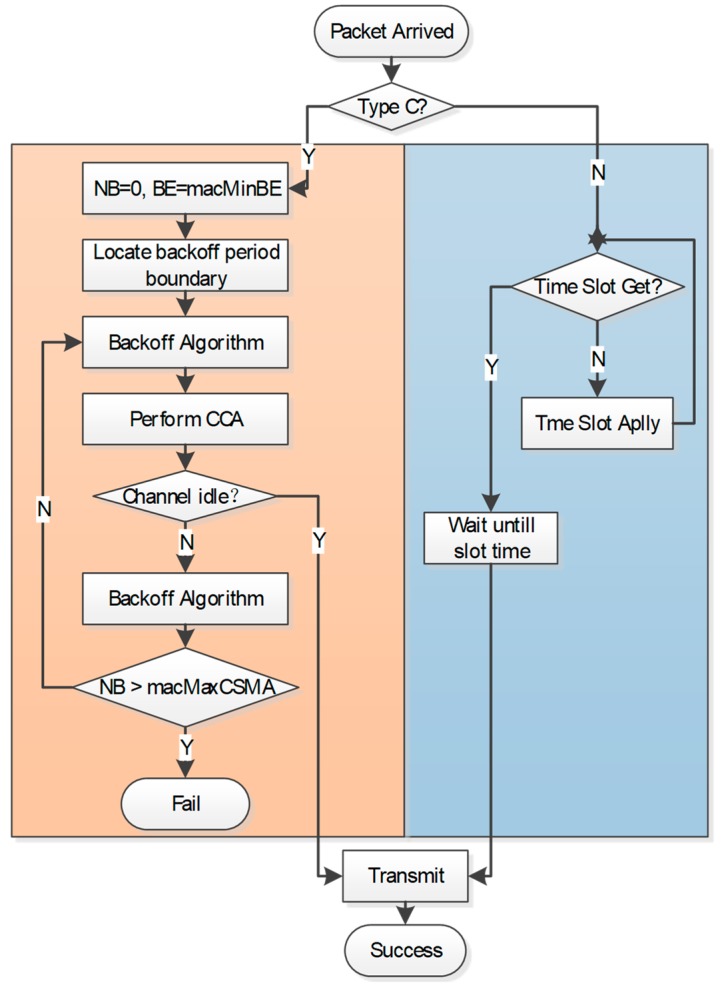
Operating process of a ISWN node.

Meanwhile, during the Extended Communication period, a slotted CSMA/CA based mechanism is employed. Nodes in type C receive their slots with a contention access mechanism, and their data transmission only performs in ECP. For a particular node, the packet transmission process is shown in Figure 6.

### 4.3. Graded Tailoring Strategy

In IEEE 802.15.4, two clear channel assessments (CCAs) will be performed before every data packet transmission. There are two situations, after the data packet or after the ACK packet, where the channel will be detected idle. In both cases, the CCA will be successfully performed. To distinguish the two cases and avoid collisions, the double-CCA mechanism is applied.

Several improved methods were proposed to achieve better performance [41,42,43,44]. In this article, we focus on frame tailoring and frame adapting, aiming to reduce the overhead caused by CCA [41,42]. They are expected to reduce the power consumption and improve the network throughput. The heart of the strategies is to adjust the tail size of the data packet, and reserve enough time for the turnaround action, so that the ACK packet is transmitted at the next backoff boundary. This way, the existence of a complete unit before the ACK packet will be avoided, then only one CCA is required. In the frame tailoring strategy proposed by Choi [41], by padding the valid data with zeros, the physics layer data unit (PPDU) occupies a fixed length in the last backoff unit and reserves enough time for node turn around (Tx-Rx or Rx-Tx). In contrast, in frame adapting proposed by Ranjeet [42], a threshold of the data packet tail length was introduced. Similarly, the threshold ensures that there is always enough time to perform the ACK packet in the coming backoff unit. When the packet tail length exceeds the given threshold, bytes will be chopped and transmitted in next packet. 

The packet adapting strategy was considered to have a better performance in packet delay and energy consumption saving, while their throughput improvement was nearly identical. However, in frame adapting, the improved packet delay performance obtained within the chopped bytes were not taken into account. Since the chopped bytes were expected to be transmitted in the next data packet of the same node, it may lead to extra packet transmission and intensify the channel contention. Thus, we propose an improved tailoring strategy named Graded Tailoring Strategy. The improved strategy is expected to reduce the adding of the valid bytes. We assume that a same backoff unit size is used, and each backoff unit includes 10 bytes or 20 symbols. Like tailoring strategy, zeros are padded to the data packet. However, the packet tail will be filled with multiple sizes instead of a uniform one. To simplify, we put forward a two grades of packet size tailoring, and the partition point is *N* (0<X≤8) and 8 (in symbols). Assuming that the size of a data packet tail is x, if N<X≤8, then zeros will be padded to the packet and make the tail size to 8; if 0<X≤N or 8<X≤20, then zeros will be padded to make the tail size N. The strategy is demonstrated in Table 3.

**Figure 7:**
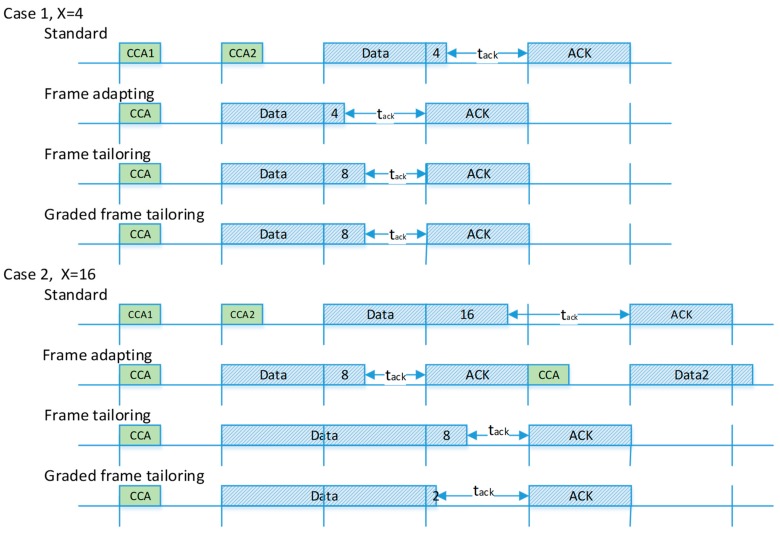
Graded Tailoring Strategies.

Note that when x is larger than 8, the new packet tail will extend to the next backoff unit. Also, assuming that the length of the valid data packet is randomly distributed, the ideal value of *N* is zero in order to reduce the invalid bytes. Besides, in CCA-ED mechanism, the channel energy checking last for eight symbols, here we assume that for energy detect, at least two symbol of data transmitting are necessary to ensure that the channel is assessed busy. Then the value of N is set to 2. The access processes of the standard strategy and the three improved strategies are shown in Figure 7.

Let *x* denote the size of the data packet tail, and there are two cases to consider: (1) 2 < x ≤ 8, and (2) 8 < x ≤ 20 or 0 < x ≤ 2, noting that they represent a continuous region. We use 4 and 16 to represent the two cases. For case 1, x = 4, the frame adapting performs best since its tail size is minimum due to the fact that it is transmitted without modification. The other two methods pad their data frame with the same length of invalid bytes. As for case 2, the tail of the frame adapting packet is chopped into two parts. The chopped bytes will cause another data transmission, leading to worse performance. For a graded tailoring strategy, the multiple tail size method avoids unnecessary invalid bytes and performs best.

To sum up, compared with the frame adapting strategy, the graded tailing avoids extra data transmission, and performs better with a probability of 75% (except 0 < x ≤ 8). When compared with a frame tailoring strategy, the graded tail padding reduces the length of invalid bytes. The proposed strategy is either equal (2 < x ≤ 8) to or better (8 < x ≤ 20 and 0 < x ≤ 2) than the packet tailoring strategy. Assuming that the length of the valid data packet is uniformly randomly distributed, the invalid bytes have dropped by 42% in the proposed method. Therefore, we have proved that the proposed strategy performs better than the two existing CCA reduction strategies.

### 4.4. Adaptive Slot Allocation Method

As mentioned earlier, the ISWN is expected to achieve both strict delay performance of partial nodes and overall operating efficiency. To further achieve energy savings, an inactive period is included in the superframe. The beacon indicates the superframe boundary, while the sub-beacons separate the different periods. Note that the TDMA period has a fixed length, while the length of CSMA period is adaptive. Thus the second sub-beacon changes with traffic load.

**Figure 8:**
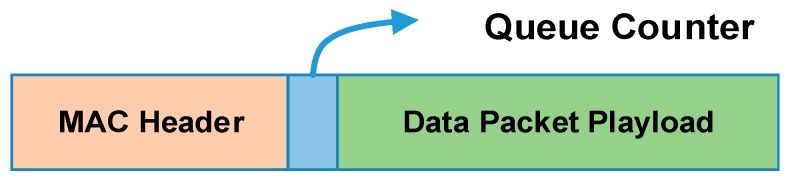
Data packet structure of ISWN.

As shown in Figure 8, in order to gather the traffic status, a queue counter is added at the beginning of the data payload. For a slave node, the queue counter carries the number of the data packets in its buffer zone. With the queue information carried by data packets, the host node can calculate the real-time network traffic. Also, in the host node, an array F[M][N] is maintained to store the slot allocation in the TDMA period, in which *M* represents the number of the slots and *N* the nodes. The value of F[m][n] (1 *< m < M*, 1 *< n < N*) represents the slot occupation status. If the slot *m* is occupied by nodes *n*, then the value of F[m][n] will be 1, otherwise it will be 0. Obviously, the sum of the elements value in the array will be less than or equal to *M*. By checking array *F*, the slot that vacated by the sleeping node is known to the host node. In this section, an adaptive slot allocation method is proposed, which includes two basic strategies. It is designed such to mantian good performance under different traffic load. The strategies are described as follows.

#### 4.4.1. Dynamic Slot Borrowing

**Figure 9:**
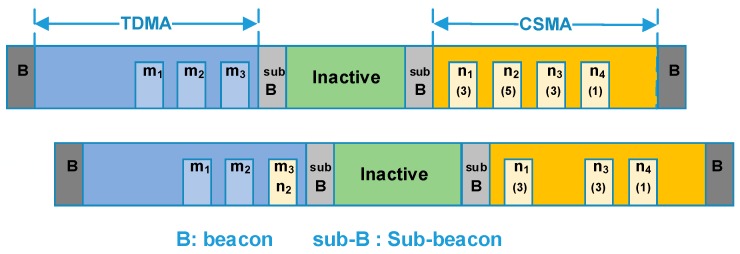
Process of dynamic slot borrowing.

As mentioned earlier, a roll-call access is adopted in the TDMA period. As shown in Figure 9, we assume that there are several nodes in the sleep mode and their slots are in idle. The last idle slot in the TDMA period (closest to the first sub-beacon) is marked as *m*_3_.

As shown in Figure 9, in the TDMA period, slots *m*_1~4_ are idle. At the same time, in the CSMA period, nodes *n*_1~4_ generate large amount of data packets. Due to the intense contention, packets are queued in their buffer zone and recorded in the queue counter. When the data packets are received by the host node, queue counters are extracted. Let q_i_ denote the list of the queue counter value, the node *n*_2_ that with the largest queue counter will be chosen, and then the idle slot *m*_3_, the slot closest to the sub-beacon, will be ‘lent’ to it. In other word, the node *n*_2_ temporarily becomes a node in the TDMA period. To avoid unnecessary control overhead, the available slots are limited, let *N_borrow_max_* be the maximum of the slots number that can be borrowed.

#### 4.4.2. Traffic Adaptive CSMA Slots Division

Since there are limited numbers of slots that can be borrowed in the TDMA period, the former strategy is sufficient for burst traffic of a few nodes. However, when the traffic of the entire network increases and data packets are queued in many nodes, additional actions are required to relieve the contention. Here, an adaptive CSMA slot division algorithm is proposed. In this algorithm, the queue counters were extracted and used to calculate the network traffic. Also, we assume that the acceptable average queue value is known to the network designer and it is marked as *Q* here. When the average value of the queue counter exceeds Q, the length of the CSMA period will increase exponentially. Otherwise, when the received data packets show that all the packet queues are zero, meaning the network traffic is light, the length of the CSMA period will decreases linearly. In extremis, all the inactive slots are divided to the CSMA period.

**Figure 10:**
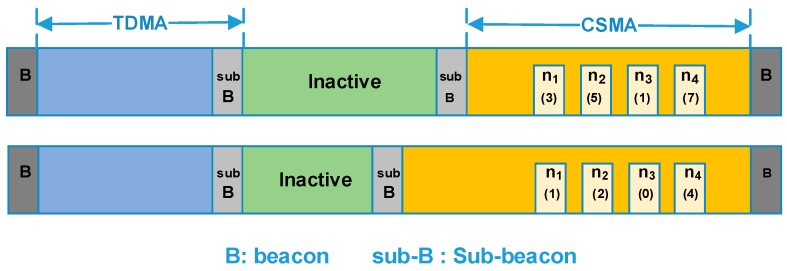
Traffic adaptive CSMA period division.

As illustrated in Figure 10, in the CSMA period, data packets are queued in several nodes. With the queue information in the received packets, the host node calculates the average value of the queue counters. Then, in the next superframe, more slots are divided to the CSMA period to relieve the heavy traffic. The algorithm is demonstrated in Table 4.

## 5. Markov Model and Calculations

### 5.1. Markov Model

To quantitatively assess the proposed protocol, especially the CSMA period, a Markov model is proposed in this section. Note that the transmission under unsaturated traffic is considered. Two stochastic processes are defined, in which S(t) represents the backoff stage and w(t) represents the backoff counter.

**Figure 11:**
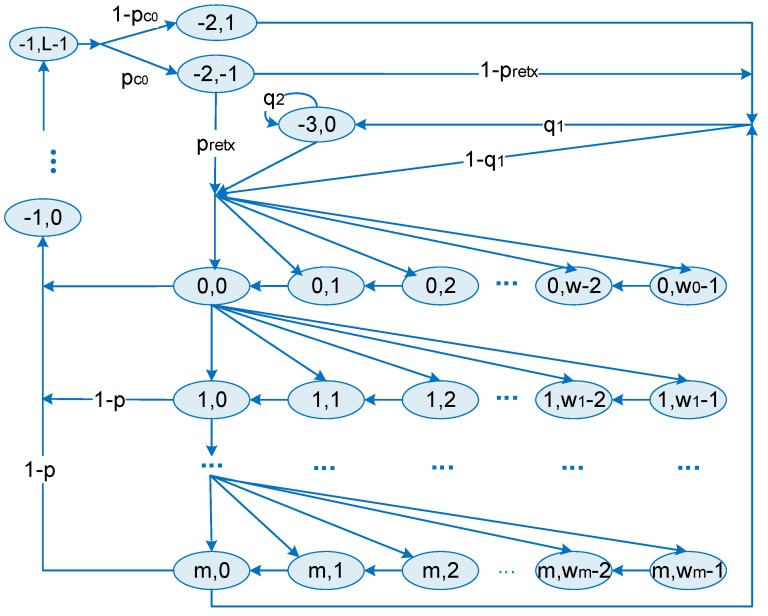
Markov model of the CSMA process.

As shown in Figure 11, the stochastic processes are divided into four states, backoff state (*s*(*t*) ≥ 0), transmission stage (*s*(*t*) = −1), and ACK state (*s*(*t*) = −2) and idle state (*s*(*t*) = −3). Then we obtain the backoff window length:

(1) $$W_{i}=2^{\min(aMaxBE,\;MinBE+i)}W\;i\;\in \;\left(0,\;m\right)$$

The transition probabilities of the Markov chain can be subsequently derived as follows:

(1) During the backoff process, the backoff counter decreases when a slot arrives:
  (2) $$p\left\{i,k|i,k+1\right\}=1\;i\;\in \;\left(0,\;m\right),\;k\in (0,\;w_{i}-2)$$
(2) When the value of backoff counter reaches zero, a clear channel assessment (CCA) is performed. Data packets will be sent if CCA success. Let p be the probability of a channel being occupied:
  (3) $$\left\{ \begin{array}{ll} p\left\{-1,0|i,0\right\}=1-p & i\;\in \;\left(0,\;m\right)\\ p\left\{-1,k|-1,k-1\right\}=1 & k\;\in \;\left(1,\;G-1\right) \end{array}\right.$$
(3) If the channel is occupied and CCA fails, the backoff stage increases and the value of backoff counter will be randomly chosen:
  (4) $$p\left\{i,k|i-1,0\right\}=p/w_{i},\;i\;\in \;\left(1,\;m\right),\;k\in (0,\;w_{i}-1)$$
(4) If a packet is successfully received, an ACK will be sent. Otherwise, the lack of ACK indicates a node collision event. Let $p_{c_{\circ }}$ denote the probability of node collision:
  (5) $$\left\{ \begin{array}{l} p\left\{-2,1|-1,G-1\right\}=1-p_{c_{\circ }}\\ p\left\{-2,-1|-1,G-1\right\}=p_{c_{\circ }} \end{array}\right.$$
(5) After a packet transmission, the backoff process will be restarted if the data buffer is not empty. Otherwise, the node will transit to the idle state:
  (6) $$\left\{ \begin{array}{l} p\left\{0,k|-2,1\right\}=(1-q_{1})/w_{0},\;k\in (0,\;w_{0}-1)\\ p\left\{-3,0|-2,1\right\}=q_{1} \end{array}\right.$$
(6) When a collision occurs, the packet will be retransmitted unless it reaches the retransmission limit  R. In that case, it will be transited to the idle state or restart the backoff process with the same probability distribution in a successful transmission. Let $p_{retx}$ denote the probability of retransmission, then we have:
  (7) $$\left\{ \begin{array}{l} p\left\{0,k|-2,-1\right\}=p_{retx}+(1-p_{retx})(1-q_{1})/w_{0}\;k\in (0,\;w_{0}-1)\\ p\left\{-3,0|-2,-1\right\}=q_{1}(1-p_{retx}) \end{array}\right.$$
(7) At the end of the backoff process, when the backoff stage reaches its maximum, if the channel is still occupied, the transmission will be cancelled. Later, if new packets arrive, the backoff process will be restarted. Otherwise, the node becomes idle:
  (8) $$\left\{ \begin{array}{l} p\left\{0,k|m,0\right\}=p(1-q_{1})/w_{0},\;k\in (0,\;w_{0}-1)\\ p\left\{-3,0|m,0\right\}=p\cdot q_{1} \end{array}\right.$$
(8) During the idle state, if a new packet arrives, the backoff process will be restarted. Otherwise, the node will remain idle. Let $q_{2}$ denote the probability of having no new packet, then we have:
  (9) $$\left\{ \begin{array}{l} p\left\{0,k|-3,0\right\}=(1-q_{2})/w_{0},\;k\in (0,\;w_{0}-1)\\ p\left\{-3,0|-3,0\right\}=q_{2} \end{array}\right.$$

Let $b_{i,\;k}$ be the probability of the stationary process, and {*i*, *k*}, (i.e., $b_{i,\;k}=\;\mathrm{limt}\to \infty \;P\;\left\{s\left(t\right)\;=\;i,\;c\left(t\right)\;=\;k\right\}$), obviously. We have:

(10) $$\begin{array}{ll} b_{i,k}=\frac{w_{i}-k}{w_{i}}b_{i,0} & i\;\in \;\left(1,\;m\right),\;k\;\in \;\left(0,\;w_{i}-1\right)\\ b_{i,0}=p^{i}b_{0,0} & i\;\in \;\left(1,\;m\right)\\ b_{-1,k}=(1-p)\sum_{i=0}^{m}b_{i,0} & k\;\in \;\left(0,\;G-1\right)\\ b_{-2,k}=\left\{ \begin{array}{l} (1-p_{c_{\circ }})b_{-1,L-1}\\ p_{c_{\circ }}b_{-1,L-1} \end{array}\right. & \begin{array}{l} k=1\\ k=-1 \end{array}\\ b_{-3,0}={\frac{q_{1}}{1-q}}_{2}(1-p_{c_{\circ }}p_{retr}(1-p^{m+1}))b_{0,0} & \end{array}$$

According to the normalize of the transition probabilities, we can get:

(11) $$\sum_{i}^{}\sum_{k}^{}b_{i,k}=1,$$

(12) $$b_{-3,0}+b_{-2,1}+b_{-2,-1}+\sum_{k=0}^{G-1}b_{-1,k}+\sum_{i=0}^{m}\sum_{k=0}^{w_{i}-1}b_{i,k}=1$$

Then we can have:

(13) $$b_{0,0}=\frac{2(1-q_{2})(1-p)(1-2p)}{P^{\prime }},$$

where:

(14) $$\begin{array}{l} P^{\prime }=2q_{1}(1-p)(1-2p)(1-p_{c_{\circ }}p_{retr}(1-p^{m+1}))\\ \;+2(G+1)(1-q_{2})(1-p)(1-2p)(1-p^{m+1})\\ \;+(1-q_{2})(1-2p)(1-p^{m+1})\\ \;+2^{MinBE}(1-q_{2})(1-p)(1-{\left(2p\right)}^{m+1}) \end{array}$$

For a single device, the probability of performing CCA is:

(15) $$\tau =\sum_{i=0}^{m}b_{i,0}=\frac{\left(1-p^{m+1}\right)}{\left(1-p\right)}b_{0,0},$$

We defined p as the probability of the channel being busy:

(16) $$p=(G+L_{ack}(1-P_{c_{*}}))(1-{\left(1-\tau \right)}^{n-1})(1-p)$$

### 5.2. Transmission under Unsaturated Traffic

For a particular node, the operating probability of the backoff process depends on the average packet arrival rate. According to existing research [31,45], we assume that the process obeys the Poisson Distribution. $T_{s}$ is the average service time, and let $q_{1}$ and $q_{2}$ are probabilities of turning to idle state or maintaining in this state after data transmission, respectively. Then we have:

(17) $$q_{1}=e^{-\lambda T_{s}},\;q_{2}=e^{-\lambda W},$$

where the $T_{s}$ consists of two parts, transmission and backoff stages:

(18) $$\left\{ \begin{array}{l} T_{s}=T_{tran}+T_{no\_tran}\\ T_{tran}=\sum_{i=0}^{m}p^{i+1}(G+1+\sum_{j=0}^{i}\frac{W_{j}-1}{2}+i)\\ T_{no\_tran}=p^{m+1}\sum_{j=0}^{m}\left(\frac{W_{j}-1}{2}+1\right) \end{array}\right.,$$

where:

(19) $$T_{tran}=\sum_{i=0}^{m}p^{i+1}(G+1+\sum_{j=0}^{i}\frac{W_{j}-1}{2}+i),$$

(20) $$T_{no\_tran}=p^{m+1}\sum_{j=0}^{m}\left(\frac{W_{j}-1}{2}+1\right)$$

### 5.3. Calculations

In this section, several main parameters of the network are calculated. Without specification, parameters are defined to describe the contention access process during the CSMA period. To avoid confusion, at the end of this article, Table A1 is given and detailed description of the notations can be found.

(A) Throughput

Let *N_slots_* denote the number of the slots needed for a successful transmission, and let *L* be the length of the packet. We then have:

(21) $$N_{slots}=nL\tau {\left(1-\tau \right)}^{n-1}p$$

Here we define $T_{CSMA}$ as the throughput of the CSMA period of the network:

(22) $$T_{CSMA}=n\tau {\left(1-\tau \right)}^{n-1}p$$

As mentioned earlier, the superframe of the hybrid network consists of three periods, and there beacons are included, while there are no packets transmission during the inactive period. Let $T_{TDMA}$ and $T_{CSMA}$ be the throughput of the TDMA and CSMA period, and the $L_{TDMA}$, $\;L_{CSMA}$ be the lengths they occupy, then we obtain the throughput of the entire network $T_{*}$:

(23) $$T_{*}=\frac{L_{TDMA}T_{TDMA}+L_{CSMA}T_{CSMA}}{L_{TDMA}+L_{CSMA}+L_{Inactive}+3}$$

(B) Transmission Probability $T_{c}$

For a particular node, transmission happens only when the channel is idle and the node performs a CCA. Then we define its transmission probability:

(24) $$P_{tx_{\circ }}=L\tau (1-p)$$

For the CSMA period, the transmission probability will be:

(25) $$P_{tx_{*}}=L\tau (1-{\left(1-\tau \right)}^{n})(1-p)$$

(C) Collision probability

For a single node, the per-node collision occurs when the node is in CCA, while there are other nodes performing the CCA as well. We define probability of such collision as $P_{c_{\circ }}$. Then we get:

(26) $$P_{c_{\circ }}=1-{\left(1-\tau \right)}^{n-1}$$

For the whole CSMA period, a collision occurs when one node is transmitting, while there are other transmitting nodes at the same time. Then we have the collision probability $P_{c_{*}}$.

(27) $$P_{c_{*}}=1-\frac{n\tau {\left(1-\tau \right)}^{n-1}}{1-{\left(1-\tau \right)}^{n}}$$

(D) Packet discard probability

For a particular attempt, Let ${\bar{\;p}}_{suc}$, $p_{col}$ and $p_{suc}$ denote the probabilities of failure, collision and success, respectively, and we have:

(28) $$\left\{ \begin{array}{l} p_{fail}=p^{m+1}\\ p_{col}=p_{c_{\circ }}(1-p_{fail})\\ p_{suc}=(1-p_{c_{\circ }})(1-p_{fail}) \end{array}\right.$$

There are two reasons for a packet to be discarded: collision and channel access failure. We define $p_{d}$ as the probability of the packet discard, and $\;p_{dc}$, $p_{df}$ as the probability of packet discard due to collision and failure. We can derive:

(29) $$p_{d}=p_{dc}+p_{df},$$

where:

(30) $$p_{dc}=p_{col}^{R+1},$$

and:

(31) $$p_{df}=\sum_{i=0}^{R}p_{fail}p_{col}{}^{i}\;=\;p_{fail}\frac{1-p_{col}^{R+1}}{1-p_{col}^{}},$$

Then we have:

(32) $$p_{d}=p_{dc}+p_{df}=p_{col}^{R+1}+p_{fail}\frac{1-p_{col}^{R+1}}{1-p_{col}^{}}$$

(E) Retransmission Probability

For a particular node, no ACK will be sent or received when collision happens. In this case, retransmission will be executed unless the packet has been retransmitted *R* times, in which *R* represents the maximum times of retransmission. We have the probability of retransmission when a collision happens:

(33) $$p_{retx}=1-\frac{p_{retx}^{R}}{\sum_{i=0}^{R}p_{retx}^{i}}=1-\frac{{\left((1-p_{fail})p_{c_{\circ }}\right)}^{R+1}}{\sum_{i=0}^{R}{\left((1-p_{fail})p_{c_{\circ }}\right)}^{i}}$$

(F) Power Consumption

Due to the limited power of an on-orbit satellite, the power consumption of the wireless network should be given more attention. Let ${\bar{E}}_{CSMA}$ be the average energy consumption during the contention period, and let ${\bar{n}}_{B}$ and ${\bar{n}}_{C}$ be the average slot number required in backoff and CCA for each attempt. Also, let $\;P_{rx}$, $P_{tx}$ and $\;P_{id}$ denote the power consumption in packet receiving, transmitting and idle sate. Then the average power consumption ${\bar{E}}_{CSMA}$ can be given as:

(34) $${\bar{E}}_{CSMA}=\frac{{\bar{n}}_{B}E_{id}+{\bar{n}}_{C}E_{rx}+(1-p_{fail})(E_{id}+E_{rx})+LE_{tx}}{{\bar{n}}_{B}+{\bar{n}}_{C}+(2+L)(1-p_{fail})}$$

(G) Delay

Consider the average packet delay in the contention period, let $\bar{n}B_{tx}$, $\bar{n}C_{tx}$ the average number of slots consumed in the backoff and CCA stages, and ${\bar{r}}_{suc}$ represents the average retransmission times, let $\bar{D}$ the average number slots needed for a success transmission, then we have:

(35) $${\bar{D}}_{CSMA}=(\bar{n}B_{tx}+\bar{n}C_{tx}+L+2)({\bar{r}}_{suc}+1)-2$$

where:

(36) $${\bar{r}}_{suc}=\sum_{i=0}^{R}i\frac{p_{suc}p_{col}^{i}}{1-p_{d}}=p_{col}\frac{1-(R+1)p_{col}^{R}+Rp_{col}^{R+1}}{\left(1-p_{col}^{R+1})(1-p_{col}\right)}$$

(H) Related Parameters

In any backoff stage, if the CCA finds that the channel is idle, the transmission will be started. The mean number of backoff for successful transmission can be derived:

(37) $${\bar{n}}_{B_{tx}}=\sum_{k=0}^{m}\left(\sum_{k=0}^{i}\frac{W_{k}-1}{2}\right)\frac{p_{s_{i}}}{1-p_{fail}},$$

where $p_{s_{i}}$ denote the probability that in backoff stage *i*, the CCA performed successfully, then we have:

(38) $$p_{s_{i}}=(1-p)p^{i}\;i\in (0,m)$$

Also, we obtain the average backoff number for fail attempts:

(39) $${\bar{n}}_{B_{f}}=\sum_{k=0}^{m}\left(\frac{W_{k}-1}{2}\right)\frac{p_{S_{m}}}{p_{fail}}=\sum_{k=0}^{m}\frac{W_{k}-1}{2}$$

Finally, the average backoff number consumption in an attempt is:

(40) $${\bar{n}}_{B}={\bar{n}}_{B_{tx}}(1-p_{fail})+{\bar{n}}_{B_{f}}p_{fail}$$

Similarly, the average time of CCA includes the successful access and failure:

(41) $${\bar{n}}_{C}=\;{\bar{n}}_{C_{tx}}(1-p_{fail})+{\bar{n}}_{C_{f}}p_{fail}$$

Since the node only performs one CCA in each backoff stage, we have:

(42) $${\bar{n}}_{C_{f}}=m+1,$$

(43) $${\bar{n}}_{C_{tx}}=\sum_{i=0}^{m}p^{i}(1-p)i,$$

(44) $${\bar{n}}_{C}=(1-p_{fail})\sum_{i=0}^{m}p^{i}(1-p)i+p_{fail}(m+1)$$

## 6. Simulations and Discussion

In the previous section, a detailed Markov model and related calculations are given. In order to validate the proposed model, an OPNET-based simulation is carried out here. We mainly focus on several major parameters of the network, including the throughput, packet delay and packet discard probability. With the simulation and analytical results, the performance of the on-board network is evaluated. A star topology network is established, in which one node is set as the host node and the others slave nodes, and only single-hop communication is involved.

According to our design experience and that of other research groups [31,46], in every sampling period, the data size of a typical on-board subsystem is around 60~70 bytes. Thus, their data packet is set to 80 bytes (need 1.28 ms). The length of the superframe is 200 ms and it is divided into 100 slots (includes 3 beacons). Therefore, the length of each slot is 1.6 ms and the gap between the slots is 0.4 ms. The TDMA period contains 40 slots. We assume that there are 40 nodes in this period, which means every node receives one scheduled slot in this period. While in the CSMA period, the nodes number varies from 5 to 30, and its length is up to 57 slots. Several default values of the parameters are specified in the simulation as m=4, BE=4 and NB=2.

**Figure 12:**
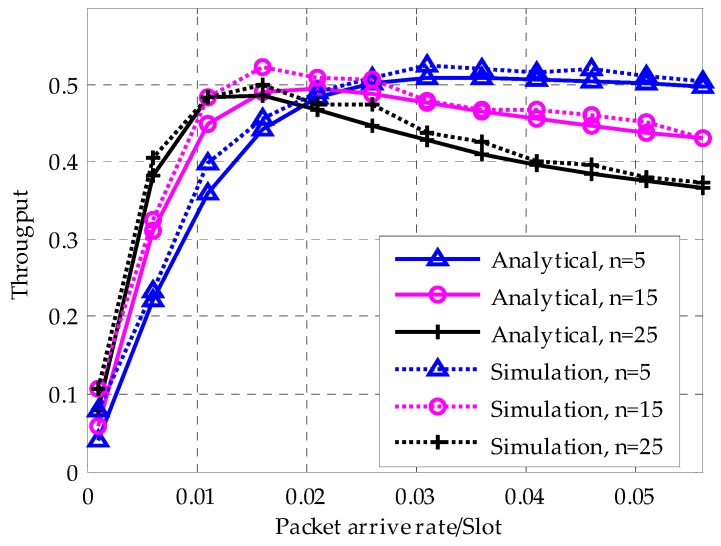
Cooperation of throughput for different network size.

Figure 12 shows the variation of throughput with respect to packet arrive rate λ and network size. With the increase of *λ*, the network throughput increases until it reaches the maximum. The growing λ then causes more frequent collisions and the throughput consequentially falls with it. For different network sizes, the larger networks have a higher throughput in small λ and they reach their maximums faster. However, since larger networks suffer more from collisions, their throughputs are declining more rapidly, too.

**Figure 13:**
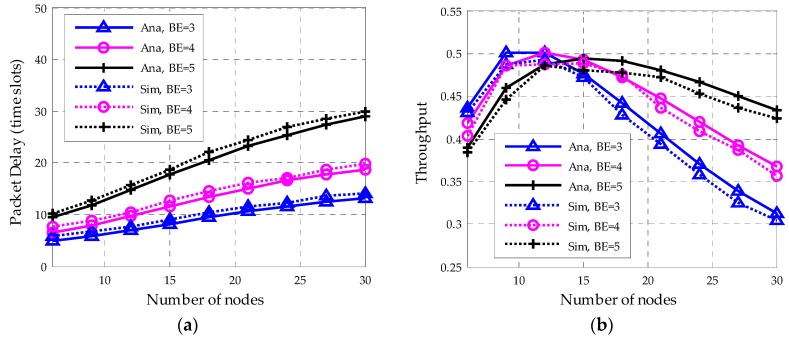
The variation of packet delay and throughput with nodes number for different BE value. (**a**) Average packet delay; (**b**) Network throughput.

Figure 13a shows the variation of average packet delay with BE. The packet arrival rate is set to 0.01 per slot. With the growth of the network scale, the average packet delay increases too. Further, networks with smaller BE have better delay performance. However, as shown in Figure 13b, for a large-scale network, a lager BE relieves the collision and performs better in throughput. Therefore, when there are few nodes in the network, it is more appreciate to choose a small BE since the collisions happen in a low probability. In addition, a long backoff window will lead to unnecessary time consumption and jeopardize both the packet delay and network throughput. On the other hand, for a large-scale network, a long backoff window helps to relieve the collisions and achieves higher throughput.

**Figure 14:**
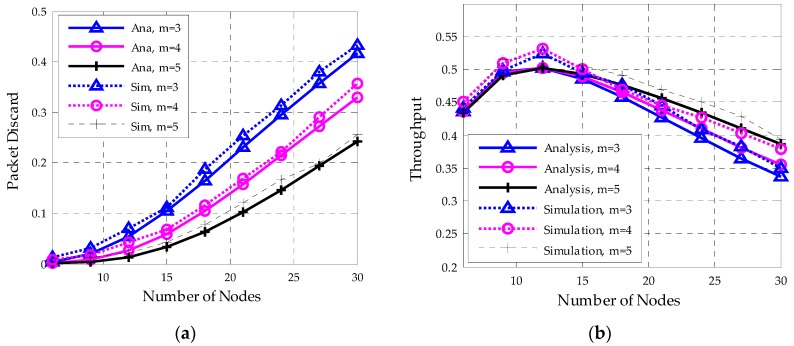
Packet delay and throughput with nodes number for different *macMaxCSMA*(*m*). (**a**) Packet discard rate; (**b**) Network throughput.

The maximum number of retransmission (*R*) is set to 3. If a data frame has been retransmitted for 3 times, then it will be directly discarded. As shown in Figure 14a, it is clear that a lager m will effectively reduce the packet discard probability. A larger *macMaxCSMA* value indicates that more backoff processes are performed and it leads to a lower transmission failure rate. At the same time, as shown in Figure 14b, when the network has very few nodes, the network performs higher throughput with a smaller *m*. With the increase of the network scale, a larger m assists to relieve collisions and achieve higher network throughput. Since the packet discard probability and the throughput are influenced differently by the *macMaxCSMA*, both the network size and performances requirements should be considered when selecting a suitable *macMaxCSMA* value.

**Figure 15:**
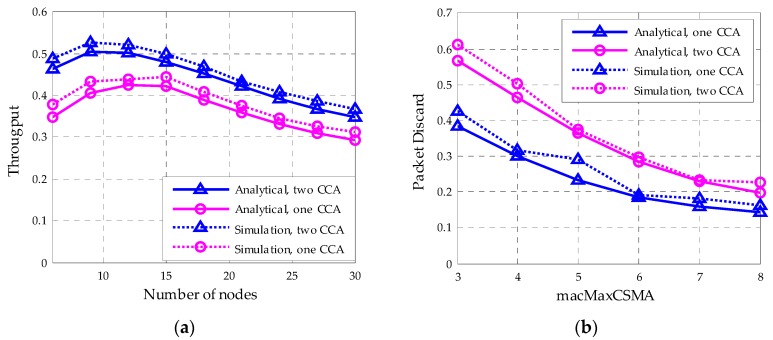
Comparison of network throughput and packet discard probability for one CCA and two CCA. (**a**) Network throughput; (**b**) Packet discard rate.

As shown in Figure 15a, the one CCA strategy adopted in this article obtains higher throughput than the standard two CCA strategies. Also, as shown in Figure 14b, when the nodes number is 25 and the packet arrive rate is set to 0.02 per slot, in both cases, with the increase of *macMaxCSMA*, the packet discard probability shows continuous decay. The proposed protocol also achieves a lower packet discard rate.

**Figure 16:**
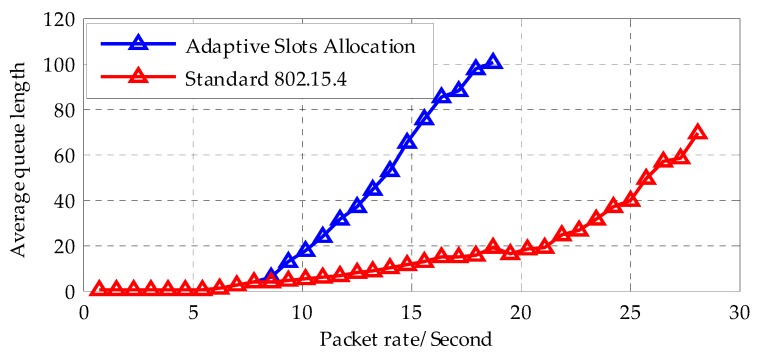
Comparison of average queue length for ISWN and 802.15.4.

Figure 16 shows the average queue delay in the network. When the packet rate is less than nine packets per second, the network maintains a few queues in both schemes. With the increase of the packet arrive rate, in the standard 802.15.4; the average queue length grows rapidly. In contrast, in the traffic adaptive slot allocation algorithm, the average queue length grows slowly until the packets rate reaches 20 packets per second. Therefore, the proposed method can effectively release the queue accumulation and tolerate a heavier traffic.

## 7. Conclusions

In this work, an intra-satellite wireless network (ISWN) for ORS satellites is proposed. A COTS- based hardware and modularized structure is constructed, which effectively reduces the volume and weight of the satellite platform. Then, flexible design and cost reduction in satellite design and launching can be achieved. A hybrid TDMA/CSMA protocol is proposed, in which two basic mechanisms—an improved single CCA strategy and a traffic adaptive slot allocation method—are proposed and verified. A Markov model is established and a detailed calculation is given to quantitatively analyze the practical design issues. Simulation experiments are carried out, and the effects of several main parameters on network performance are identified. The simulation results indicate that the proposed hybrid protocol outperforms the existing schemes both in capacity and packet discard rate. Also, through the traffic adaptive slot division, both energy saving and performance guarantee are achieved. The proposed scheme is proven to meets the on-orbit data demands and will be a promising candidate to replace the conventional wired data bus. In future work, we will focus on the layout and shield design. Semi-physical simulation and on-orbit verifications also need to be carried out.

## Figures and Tables

**Table 1:** Parameters of the wireless nodes.

Parameters	Value	Units
**Micro Processor**		
Program memory	64	KB
RAM	4352	Byte
Active Power	27	mW
Sleep Power	27	μW
Storage Size	64	KB
**RF transceiver**		
Data rate	500	Kbps
Receive power	40	mW
Transmit power at 0 dBm	68	mW
**ISWN Node**		
Total Active power	143	mW

**Table 2:** On-board data classification.

Type	Data Rate	Delay	Examples
A	High	Sensitive & insensitive	Payloads, on-board computer, telemetering and tele-control system, power management system, etc.
B	Low	Sensitive	Thrusters, reaction wheels, GPS, gyroscope, magnetometer, star camera, Sun sensors, etc.
C	Low	Insensitive	Thermal subsystem, mechanical subsystem temperature sensors, solar panel and other sensors

**Table 3:** Graded Tailoring Strategy.

Graded Tailoring Strategy
X: the original length of the data packet in the last unit Y: the size of the packet tail after the graded tailoring N: the partition point Check the length of the physics layer data unit (PPDU) If N<X≤8 Y←8 else Y←N Stop

**Table 4:** Traffic adaptive CSMA slot division algorithm.

Adaptive Slots Allocation Method
K: Slots division index $N_{borrow\_max}$: Maximum of borrowed slots $N_{idle}$: Number of idle slots F[M][N]: Slots occupation matrix in the TDMA period S(i): List of idle slots in the TDMA period q(i): Queue counter value of node *i* $\bar{q}$: Average queue counter value of received data packets Q: acceptable average queue threshold $L_{csma}$: Slots number in the CSMA period entry 1
**Start** Collect the data packets in the CSMA period and calculate K←1 For $i\;\leftarrow 1\;to\;N_{borrow\_max}$ refresh F[M][N] Count the number of idle slots $N_{idle}$ and find the last idle slot S(r) Sort q(i) and find the largest value q(j) If $N_{idle}>0$ then F[i][j]←1 end Wait for the next superframe If $\bar{q}\geq Q$ then $$L_{max}\leftarrow \max\left(L_{max}+2^{k},L_{max}.\max\right)$$ else if $\bar{q}=0$ then K←0 $L_{csma}\leftarrow L_{csma}-2$ **Stop**

## Appendix A

In Table A1, a summary of the notations employed in this paper is given.

**Table A1:** List of notations.

Symbol	Description	Symbol	**Description**
L	packet length (in slots)	$P_{tx_{o}}$	*Pr* {one node is in transmitting}
$L_{CSMA}$	length of the CSMA period	$P_{tx_{*}}$	*Pr* {the network is in transmitting}
$L_{TDMA}$	length of the CDMA period	$P_{c_{o}}$	*Pr* {node collision}
m	number of back off stage	$P_{c_{*}}$	*Pr* {network collision}
$W_{i}$	the *i^th^* backoff window size	$P_{d}$	*Pr* {packet discard}
W	size of backoff unit	$P_{dc}$	*Pr* {packet discard due to collision}
α	*Pr* {CCA fail}	$P_{df}$	*Pr* {packet discard due to access failure}
τ	*Pr* {node is performing CCA}	$P_{suc}$	*Pr* {attempt success}
p	*Pr* {channel busy}	$P_{col}$	*Pr* {attempt and collision}
$T_{s}$	average service time for a packet	$P_{fail}$	*Pr* {access failure}
$T_{*}$	throughput of the hybrid work	$P_{retx}$	*Pr* {retransmission after collisions}
$T_{CSMA}$	throughput of the CSMA period	${\bar{D}}_{CSMA}$	average packet delay in CSMA period
$T_{TDMA}$	throughput of the TDMA period	${\bar{E}}_{CSMA}$	average power consumption in CSMA period
